# Basic and clinical immunology – 3018. Immunocap vs. skin prick testing for inhalant allergens

**DOI:** 10.1186/1939-4551-6-S1-P194

**Published:** 2013-04-23

**Authors:** Prem Kumar, Annette Fiorillo, Chitra Desai, Anush Agrawal

**Affiliations:** 1 Louisiana State University Health Sciences Center, Department of Medicine. Section of Allergy and Clinical Immunology, New Orleans LA, USA; 2 Louisiana Health Sciences Center, USA; 3 School of Medicine, Texas Tech University, Paul L Foster, El Paso, USA

## Background

Skin Prick Testing has long been considered the standard for detecting sensitization to inhalent Allergens. However, in vitro testing for specific IgE is also common practice. Various studies have shown that these two methods are equal in sensitivity and specificity, while others have indicated that in vitro testing is less sensitive. This study was performed to compare in vitro Immunocap testing to Skin Prick Testing for identifying sensitization to common southern Aero-Allergens in patients with history of Allergic Rhinitis.

## Methods

Fifteen patients (age range 25 to 48 years, 7 males and 8 females) suffering from perennial Allergic Rhinitis were studied. Eleven of fifteen patients reported worsening of Rhinitis symptoms during Spring. In addition, nine of eleven did have symptoms consistent with Allergic Conjunctivitis. Skin Prick Testing was performed in accordance with published practice parameters (Bernstein L. et al, Annals of Allergy, Asthma, Immunology, Vol. 100, #3, supplement 3, March 2008.). In addition, Immunocap Testing (Quest Diagnostics) for specific IgE was performed on Cockroach, Dust Mite, D. Farinae and D. Pteronyssinus, Grass Pollens: Bahia, Bermuda, Johnson, Timothy, Italian Rye, & Tree Pollens; Oak, Elm, Maple, Pecan, & Sycamore. The sensativity between these two methods was analyzed.

## Results

Skin Prick Test for Grass and Tree Pollens were positive in 13/15 (87%) patients. However, Immunocap Testing was positive in only two of fifteen (13%) patients for Grass and Tree Pollens. In contrast, for Dust Mite (D. Farinae, D. Pteronyssinus), both Immunocap and Skin Prick Test were positive in only three of fifteen (20%) patients. Furthermore, in one of fifteen (7%), results were positive for Cockroach; both by Skin Prick Test & Immunocap Assays.

## Conclusions

In patients with Allergic Rhinoconjunctivis, Skin Prick Tests may be more sensitive in detecting sensitization to Grass and Tree Pollens in comparison with Immunocap Testing.

